# Postmortem Stability of SARS-CoV-2 in Mouse Lung Tissue

**DOI:** 10.3201/eid2712.211621

**Published:** 2021-12

**Authors:** Sophie A. Valkenburg, Samuel M.S. Cheng, Asmaa Hachim, Malik Peiris, John Nicholls

**Affiliations:** The University of Hong Kong, Hong Kong, China

**Keywords:** SARS-CoV-2, infectious virus, antigen, RT-PCR, K18 hACE2 mice, TCID50, postmortem, mice, experimental infection, COVID-19, respiratory infections, severe acute respiratory syndrome coronavirus 2, 2019 novel coronavirus disease, coronavirus disease, zoonoses, viruses, coronaviruses

## Abstract

The infectivity of severe acute respiratory syndrome coronavirus 2 in deceased persons and organisms remains unclear. We studied transgenic K18 hACE2 mice to determine the kinetics of virus infectivity after host death. Five days after death, virus infectivity in the lung declined by >96% and RNA copies declined by 48.2%.

The safe handling and disposal of bodies of persons who have died of coronavirus disease (COVID-19) is vital for infection control. Although cremation or burial practices are mainly dictated by religious and societal customs, deaths associated with contagious illness warrant appropriate precautions. Severe acute respiratory syndrome coronavirus 2 (SARS-CoV-2), the causative agent of COVID-19, is rapidly inactivated (>2 log_10_) within hours on nonporous surfaces (*1*). In addition, several studies have detected viral RNA by reverse transcription PCR (RT-PCR) of nasopharyngeal and pharyngeal mucosal swab specimens, skin swab specimens, and tissue samples collected during autopsies at different times after death (*2*–*5*). Furthermore, infectious virus was isolated in 2 of 4 cases at 4–17 days postmortem; however, this study did not quantify virus titers to determine the loss of virus infectivity (*6*). A separate study found that infectious virus was undetectable after exhumation at 3–4 months postmortem (*7*). Overall, RNA detection by RT-PCR might not directly correlate with virus infectivity or duration of symptomatic disease.

Transgenic K18-hACE2 mice provide a surrogate model to study the kinetics of SARS-CoV-2 viral replication during infection (*8*) and after host death. In humans and K18-hACE2 mice, little evidence exists for extrapulmonary dissemination of SARS-CoV-2, except for neurotropism in younger mice, a finding that has not been demonstrated reliably in humans. We investigated the temporal decay of infectious SARS-CoV-2 in postmortem tissues of infected K18-hACE2 mice. All experimental procedures were conducted in accordance with the standards and approved by the Committee on the Use of Live Animals in Teaching and Research (approval no. 5511-20) at The University of Hong Kong (Hong Kong, China).

We infected twelve 14–20-week-old mice with 1 × 10^4^ 50% tissue culture infectious dose (TCID_50_)/25μL SARS-CoV-2 by the intranasal route. Five days later, after the mice had lost 18.8% (SD 7.77%) of their body weight, we euthanized them by ketamine/xylazil anesthesia. We wrapped each carcass in a sealable plastic bag, similar to the storage of human corpses, and stored them intact at 4°C, which is standard mortuary temperature. On days 0, 1, 5, and 14 after death, we dissected 3 carcasses and tested the lung tissue for coronavirus nucleoprotein (N) by histologic and immunohistochemistry assays (*9*) (Appendix Figure, panels A–H). We quantified infectious virus by culture (Figure, panel A) and viral RNA by RT-PCR (Figure, panel B) (Appendix).

**Figure:**
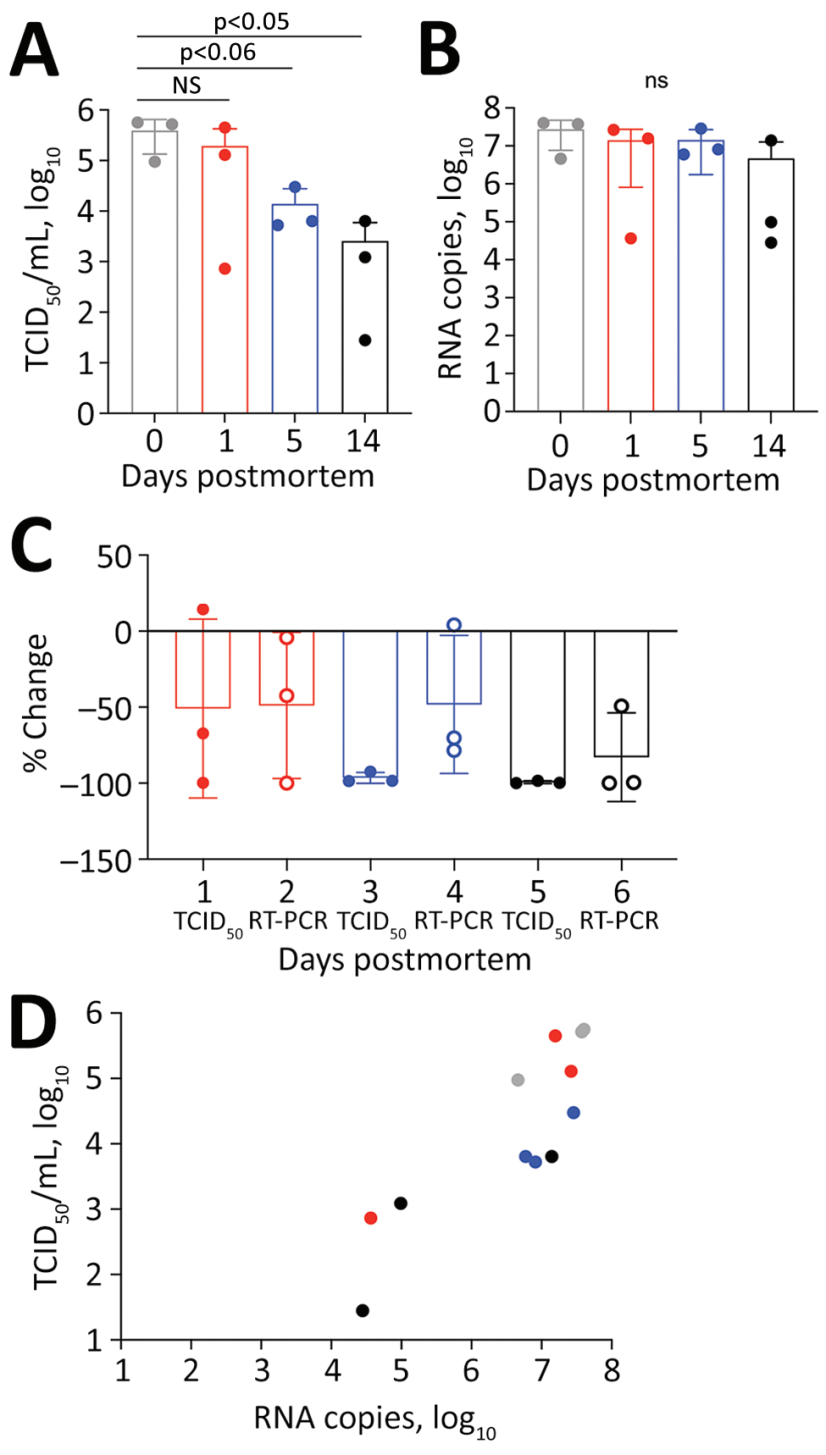
Postmortem stability of severe acute respiratory syndrome coronavirus 2 in mouse lung tissue. A) Infectious virus measured by TCID_50_ of VeroE6 cells. B) Viral RNA measured by copies of N gene detected by RT-PCR. C) Percentage change compared with day 0. D) Correlation between infectious virus and viral RNA. R^2^ = 0.51; *F* = 0.005 by analysis of variance. NS, not significant; RT-PCR, reverse transcription PCR; TCID_50_, 50% tissue culture infectious dose.

Viral decay, measured using TCID_50_ for infectious virus and RNA copies of the N gene detected by RT-PCR, occurred over a 14-day period (Table). At day 1 we observed a 50% reduction of infectious virus and 48.8% loss of viral RNA (Figure, panels A, B). By day 5, levels of infectious virus had fallen by 96.5%, whereas viral RNA remained at 48.2% compared with day 0 (Figure, panels C, D). At day 14 only 0.7% of the initial infectious virus and 17% of viral RNA remained. Plenzig et al. (*7*) detected viral RNA in 2 exhumed corpses at 3 months postmortem, despite an absence of infectious virus. We used hematoxylin and eosin staining to detect viral nucleoprotein in lung tissue. We observed persistent antigen staining until day 5; by day 14, only 1 of 3 samples had detectable staining (Appendix Figure). 

**Table:** Postmortem viral loads in K18-hACE2 mice lung tissue after 5 days of infection with severe acute respiratory syndrome coronavirus 2*

Day	N gene copies, log_10_	% Reduction of N gene copies†	TCID_50_/mL, log_10_	% Reduction of TCID_50_/mL†	Lung inflammation score‡	SARS-CoV-2 N protein antigen score§	Positive for SARS N protein¶
0	7.28 + 0.53	NA	5.48 + 0.44	NA	9.33 + 1.53	3.66 + 1.15	3 (100.0)
1	6.39 + 1.59	−48.85 + 48.14	4.54 + 1.48	−50.88 + 58.82	7 + 2	2.66 + 2.31	2 (66.6)
5#	7.05 + 0.36	−48.21 + 45.43	4.00 + 0.41	−96.48 + 3.54	5.33 + 4.61	4 + 1.4	2 (100.0)
14	5.53 + 1.43	−82.95 + 29.13	2.78 + 1.21	−99.35 + 0.86	10.33 + 1.53	1.66 + 2.88	1 (33.3)

*Values are mean +SD, except as indicated. NA, not applicable; TCID_50_, 50% tissue culture infectious dose. †Compared with day 0. ‡Scale of 0–16, in which 16 represents most severe inflammation. §Scale of 0–5, in which 5 represents highest amount of antigen. ¶Values are no. (%). #Days 0,1, and 14 values reflect 3 mice. Day 5 values reflect 2 mice for histology and 3 mice for TCID_50_ and reverse transcription PCR.

We euthanized the mice 5 days after infection, when the lungs had a high viral load. However, COVID-19 deaths usually occur during later stages of disease, by which time infectious viral load has decreased from the peak usually seen early during the symptomatic phase of the illness (*10*). We detected virus antigen in the lungs of all mice at 5 days postmortem; infectious virus had declined by 96.48%, but viral RNA declined by only 48.21%. Our results shows that infectious virus declines earlier than viral RNA or antigen in postmortem tissues.

These findings have implications for the safe handling of deceased COVID-19 patients. Infectious virus can persist on inanimate surfaces for up to 14 days at lower temperatures (<4°C), but rapidly decays in postmortem tissue samples. We observed a 96.5% decrease in infectious virus by day 5 and a 99.3% decrease by day 14. Most published postmortem studies in humans have reported viral load at the time of death using cycle threshold values rather than N gene copies as we have done; results range from 17–36 for cycle threshold values and 0–5.49 log_10_ for N gene copies (*11*). Therefore, the maximum potential risk of transmission from an infected corpse is during the first 24 hours after death. By day 5, the amount of infectious virus has decreased by 96.48%. If proper biosafety precautions and personal protective equipment are used to handle the corpse during autopsy or preparation for burial or cremation, we believe that the burial or cremation process is unlikely to spread disease.

Appendix Additional information about persistence of infectious SARS-CoV-2 in postmortem lung of experimentally infected mice.
