# Supplementary material for: Postmortem Stability of SARS-CoV-2 in Mouse Lung Tissue
Source: Emerg Infect Dis. 2021 Dec;27(12):3173–5. doi: 10.3201/eid2712.211621 (PMC8632159; doi:10.3201/eid2712.211621)
Supplement: Appendix — Additional information about persistence of infectious SARS-CoV-2 in postmortem lung of experimentally infected mice. [file 21-1621-Techapp-s1.pdf]

Article DOI: https://doi.org/10.3201/eid2712.216121

Postmortem Stability of SARS-CoV-2 in Mouse Lung Tissue

Appendix

Appendix Methods

Mouse Infection

Twelve 14–20-week-old female K18 mice expressing human angiotensin converting enzyme 2 (hACE2) (The Jackson Laboratory, https://www.jax.org) were infected at the same time with 1×10^4 $TCID_{50}$/25uL of SARS-CoV-2 (βCoV/Hong Kong/VM20001061/2020, GenBank accession no. MT547814.1) by the intranasal route. They were culled at day 5 post infection by ketamine/xylazil anesthesia.

Viral Titers

Lung viral titers were determined from homogenates by standard $TCID_{50}$ assay on VeroE6 cells, and viral titers were calculated by the Reed-Muench method as previously described (*1*). Specimens from lung homogenate supernatant were extracted using QIAamp Viral RNA kit (QIAGEN, https://www.qiagen.com) according to the manufacturer's instructions, and tested by a quantitative reverse transcription PCR selective for the N gene of SARS-CoV-2 previously published (*2*) with absolute copy number quantification. A cloned plasmid DNA extract carrying a DNA insert of the N gene was prepared and quantified for DNA copy number. The copy number control plasmid DNA with serial dilutions were included in each PCR run to construct a standard curve to correlate cycle threshold values and gene copy number of samples. Absolute N gene copies in each reaction was deduced. Virus gene copy number per mL was calculated by adjusting dilution effect by nucleic acid extraction.

Immunohistochemistry and N Antigen Staining

Lung tissues were washed in PBS and fixed at the time of collection in 10% neutral buffered formalin. They were processed for hematoxylin and eosin staining and for immunohistochemistry for SARS-CoV nucleoprotein using a rabbit polyclonal antibody (cat no.

40143-T62; Sinobiological, https://www.sinobiological.com) against SARS-CoV nucleoprotein using 1/500 dilution at room temperature for 75 min. Antigen retrieval was performed using microwave in citrate buffer (pH 6.0) for 15 min. Lung inflammation was scored according to Hovart et al. (*3*) up to a total score of 16 for inflammation in the airway (score of 0–4), vasculature (0–4), and parenchyma (score 0–5), and a separate determination of the amount of N antigen (score 0–5).

References

1. Perera RAPM, Tso E, Tsang OTY, Tsang DNC, Fung K, Leung YWY, et al. SARS-CoV-2 virus culture and subgenomic RNA for respiratory specimens from patients with mild coronavirus disease. Emerg Infect Dis. 2020;26:2701–4. PubMed https://doi.org/10.3201/eid2611.203219

2. Chin AWH, Chu JTS, Perera MRA, Hui KPY, Yen HL, Chan MCW, et al. Stability of SARS-CoV-2 in different environmental conditions. Lancet Microbe. 2020;1:e10. PubMed https://doi.org/10.1016/S2666-5247(20)30003-3

3. Horvat JC, Beagley KW, Wade MA, Preston JA, Hansbro NG, Hickey DK, et al. Neonatal chlamydial infection induces mixed T-cell responses that drive allergic airway disease. Am J Respir Crit Care Med. 2007;176:556–64. PubMed https://doi.org/10.1164/rccm.200607-1005OC

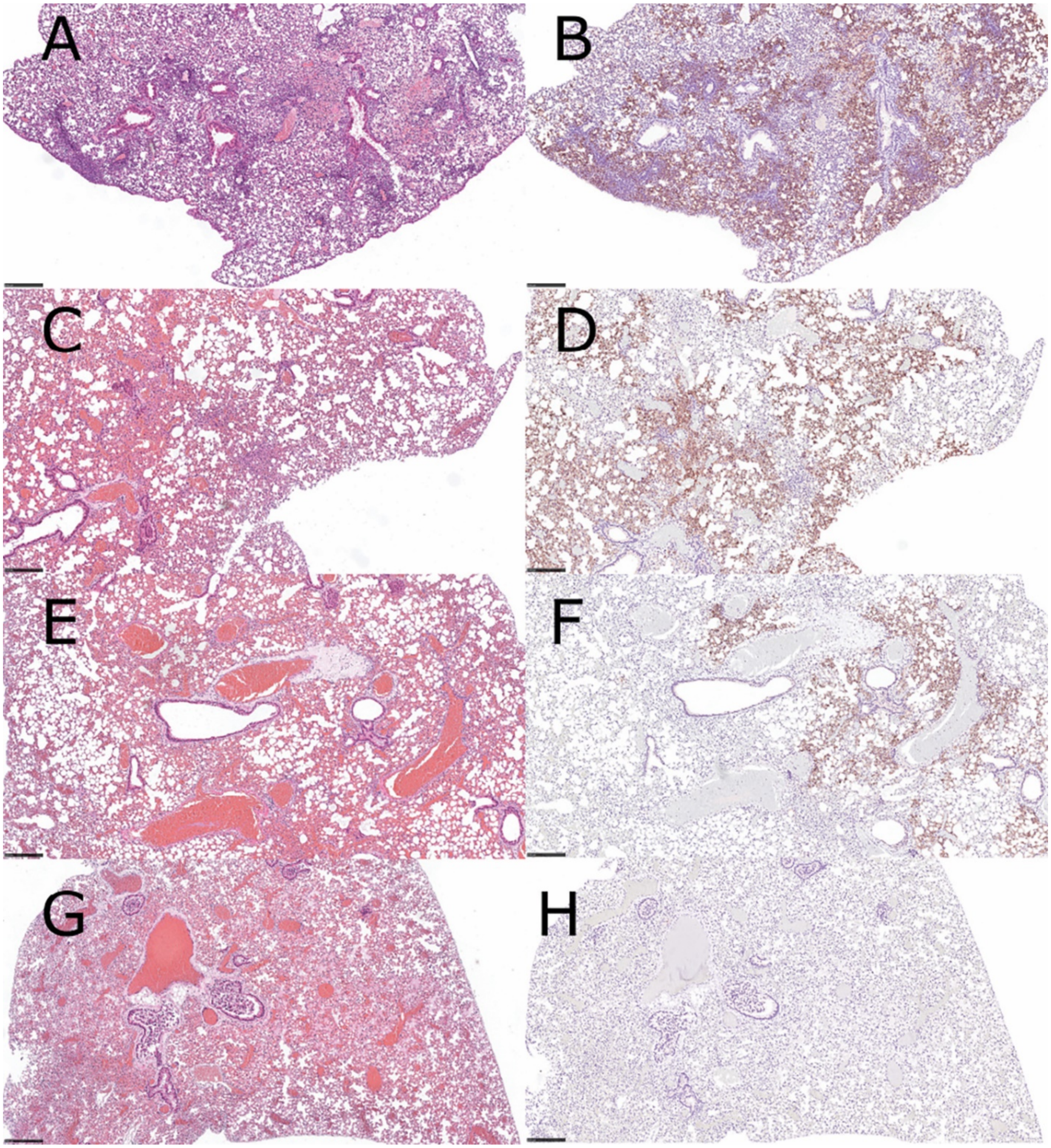

Appendix Figure. Hematoxylin and eosin staining to detect severe acute respiratory syndrome in postmortem mouse lung tissue. Lung hemorrhage and inflammation at 0 (A), 1 (C), 5 (E), and 14 (G) days after death. Decreasing amounts of antigen detected at days 0 (B), 1 (D), and 5 (F), but not 14 (H). Magnification ×50.
